# Biliary tract melanoma metastasis mimicking hilar cholangiocarcinoma: a case report

**DOI:** 10.1093/jscr/rjab549

**Published:** 2021-12-11

**Authors:** Rodrigo Piltcher-da-Silva, Vivian Laís Sasaki, Debora Oliveira Hutten, Ana Paula Percicote, Carlos Henrique Trippia, Raul Alberto Anselmi Junior, Marco Aurélio Raeder da Costa, Júlio Cezar Uili Coelho

**Affiliations:** Division of General and Digestive Surgery, Hospital Nossa Senhora das Graças, Curitiba, Brazil; Division of General and Digestive Surgery, Hospital Nossa Senhora das Graças, Curitiba, Brazil; Division of General and Digestive Surgery, Hospital Nossa Senhora das Graças, Curitiba, Brazil; Department of Pathology, Hospital Nossa Senhora das Graças, Curitiba, Brazil; Department of Radiology, Hospital Nossa Senhora das Graças, Curitiba, Brazil; Division of General and Digestive Surgery, Hospital Nossa Senhora das Graças, Curitiba, Brazil; Division of General and Digestive Surgery, Hospital Nossa Senhora das Graças, Curitiba, Brazil; Division of General and Digestive Surgery, Hospital Nossa Senhora das Graças, Curitiba, Brazil

## Abstract

Malignant melanoma is the 19th leading cause of cancer worldwide. It is an aggressive neoplastic disease in which pathophysiological understanding and management has been in constant evolution in recent decades. The primary site is the skin, uvea and mucous membranes and has the capacity to metastasize to any organ. There are few reports of primary or secondary involvement of the biliary tract. We present the case of a 73-year-old woman with a bile duct lesion suggestive of cholangiocarcinoma and a final diagnosis of a single melanoma metastasis. Surgical treatment was performed due to oligometastatic stage IV melanoma with possibility of R0 resection followed by immune checkpoint therapy.

## INTRODUCTION

Malignant melanoma (MM) has a worldwide incidence of 3.4/100 000 inhabitants and is the 19th leading cause of cancer. It is also the 22nd cause of cancer-related death for both genders worldwide [1]. The most common primary site is the skin, but in 1.3% of cases it originates from the mucous membranes of the respiratory tract, genitourinary and gastrointestinal tracts [2]. It is a multifactorial disease requiring both genetic susceptibility and environmental exposure [3].

MM metastasizes in 30–50% of cases and can affect any organ [4]. Stage IV melanoma has a 5-year overall survival (OS) of 12% for solitary metastases and 0% for multiple metastases [5]. However, discoveries of pathway-targeted inhibitors and immune checkpoint agents have modified this scenario, increasing 5-year OS-rates for metastatic melanoma to up to 40–50% [2].

Metastasis to the digestive tract (DT) occurs in only 2 to 4% of cases [2, 3, 6]. The most common site of metastasis in the DT is the small intestine (75%) followed by the colon (25%), liver (20%) and stomach (16%) [7]. The metastatic spread to the biliary system is a rare event with few reports in the literature [3, 8]. Metastasectomy is indicated only if R0 resection is possible with acceptable morbidity [2, 6]. Here, we report a case of cutaneous MM metastasis to the biliary tract.

## PRESENTATION OF CASE

Our patient is a 73-year-old female with previous medical history of well-controlled systemic arterial hypertension. The surgical history revealed ankle fracture, tubal ligation and resection of cutaneous melanoma in May 2021. Melanoma was resected in the cervical region, with anatomopathological (AP) report of nodular type, Clark IV and stage T1. A sentinel lymph node analysis was negative for neoplasia.

**Figure 1:**
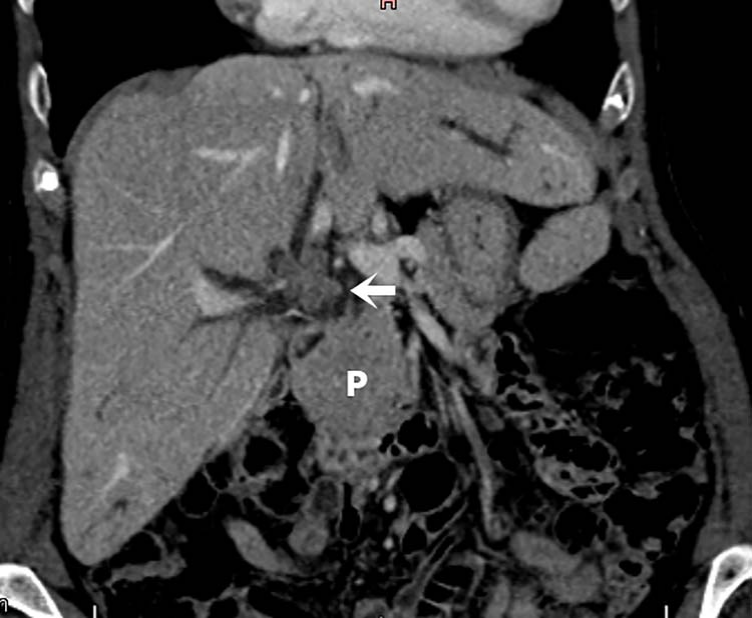
Cholangioresonance showing biliary tract dilatation due to intraductal lesion of the biliary tract, involving the confluence and the right hepatic duct (Bismuth IIIA).

**Figure 2:**
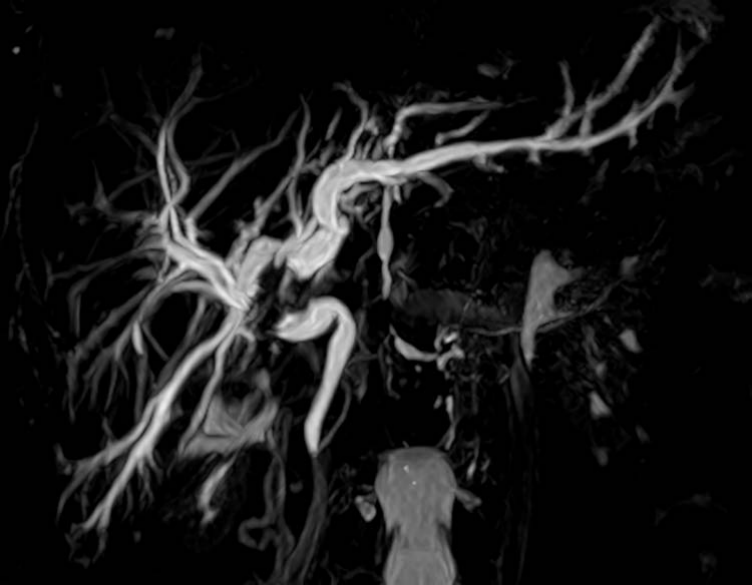
Abdominal tomography with intravenous contrast enhancement showing a solid and irregular nodule (white arrow) that caused retraction of the gallbladder bed and loss of the cleavage plane with the proper hepatic artery and portal vein, measuring 30 × 26 × 27 mm. The lesion is invading the biliary tract at the confluence of the hepatic ducts. There are no evident vascular invasion and no regional lymph node enlargement. The pancreas (P) is normal.

Screening for staging was performed, with ultrasound of the abdomen showing a suspicious neoplasia at the common hepatic duct. Cholangioresonance findings were suggestive of cholangiocarcinoma (Fig. 1). Computed tomography (CT) demonstrated a solid and irregular nodule (Fig. 2).

A right hepatectomy with resection of the common bile duct and lymphadenectomy was performed. The biliary tract was reconstructed with anastomosis of the left hepatic duct and a Roux-en-Y jejunal loop. The postoperative recovery was uneventful, with patient discharge on the sixth postoperative day.

**Figure 3:**
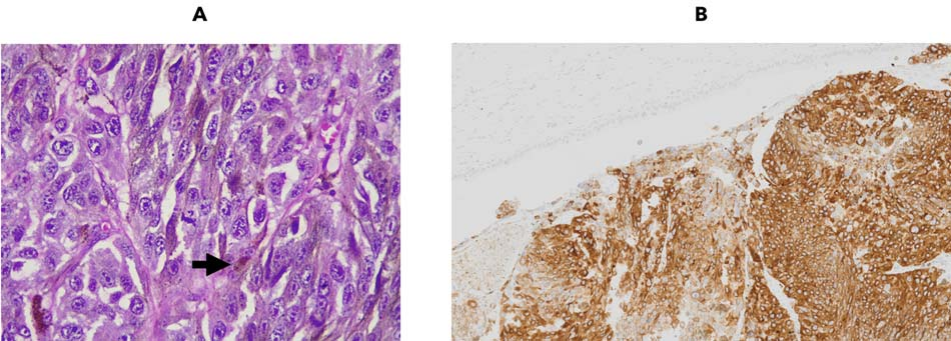
Epithelioid cells with brownish pigment between the cells (black arrow) are compromising the bile ducts from the lumen [**A**]. There is positivity for S-100, SOX-10, HMB45 and MELAN-A. Immunoexpression for MELAN-A is shown [**B**].

At operation, the aspect of the lesion and the type of involvement were consistent with the diagnosis of cholangiocarcinoma. However, after opening the surgical specimen, a brownish lesion in the biliary tract was identified. The pathologic exam demonstrated a melanoma affecting the biliary tract intraductally, with free margins and no lymph node involvement. Immunohistochemistry confirmed that was an infiltrating epithelioid melanoma (Fig. 3).

Thus, with the diagnosis of metastatic melanoma, treatment with Pembrolizumab was started. There were no metabolically active lesions on a PET/CT.

## DISCUSSION

MM is a cancer that originates from melanocytes, which derive from the neural crest (ectoderm) during embryogenesis. However, the presence of benign and malignant melanocytic populations has been demonstrated even in endoderm-derived tissues such as the gallbladder and biliary tract [9]. It can primarily affect tissues such as skin, uvea and mucous membranes, with great risk of dissemination, both through hematogenous and lymphatic system [3, 4].

They are classically divided between melanomas on sun-exposed skin and those on sun-protected skin. However, they have currently been classified according to their mutations, which is important to guide the therapy. It has been observed that in MM from exposed areas the BRAF, N-RAS and PTEN genes are the most affected. MM from unexposed areas have a greater number of chromosomal abnormalities and gene amplification, especially of CDK4 and CCND1 [10]. Proto-oncogene c-KIT mutation is present in 15% of mucosal melanomas [11].

The management of MM in stage IV has advanced in recent years. The advent of BRAF and MEK pathway-targeted inhibitors and immune checkpoint agents, such as monoclonal antibodies against cytotoxic T-lymphocyte-associated protein 4 (CTLA-4) and programmed cell death protein 1 (PD-1), has improved the OS^6^. Thus, the approach for metastatic melanoma has become increasingly complex, with a greater need to combine systemic therapy and surgery. Surgical indication in MM stage IV is based on the control of immunological factors, produced directly by tumor cells or by the adjacent tissue induced by them, which suppress the host’s antitumor immune response. With resection, immune function is restored [12, 13].

The surgical indication for the treatment of metastatic disease depends on the affected site, disease volume, possibility of R0 resection, response to systemic treatment, disease-free interval and surgical morbidity and mortality [6]. OS decreases as metastatic sites increase [13]. In R1/2 metastasectomy, the average OS is 8.4 months, against 27.6 months in cases of R0 resection [14]. Ryu et al. [4] reported 0% of 5-years OS-ratio after incomplete resection and 48% after R0 resection. However, despite the good results, only 3% of patients were candidates for resection. OS was 2–4 months for unresected hepatic disease and 28 months after R0 resection. Therefore, these data corroborate the indication of resection in our patient.

Metastases to the gallbladder and biliary tract tend to be pigmented, flat and multiple [15]. When primary, biliary tract MM still presents uncertain prognosis due to the low number of cases [3]. To differentiate between metastasis and primary biliary tract MM, the following criteria must be present: single solitary lesion, absence of previous melanoma, absence of melanoma in other sites, polypoid or papillary form and presence of biliary obstructive symptoms. However, these criteria are weak and an ultrastructural pathological assessment can help to differentiate them [9].

## CONCLUSIONS

MM is capable of hematogenous and lymphatic dissemination to any organ. During follow-up, any exam alteration should be considered a consequence of melanoma until proven otherwise. In this case, the clinical and radiological presentation was indistinguishable from cholangiocarcinoma. The resection was performed because it was indicated for both cholangiocarcinoma and metastatic melanoma.

The growing knowledge about its nature and the evolution of treatment in recent decades have improved the OS of metastatic melanoma. Multicenter studies are needed to better elucidate the prognosis of patients with isolated melanoma of the biliary tract, both in primary and metastatic disease.

## DECLARATIONS

Funding: This study did not receive any specific grant from funding agencies in the public, commercial, or non-profit sectors.

Conflict of interest: The authors Rodrigo Piltcher-da-Silva, Vivian Laís Sasaki, Debora Oliveira Hutten, Ana Paula Percicote, Carlos Henrique Trippia, Raul Alberto Anselmi Junior, Marco Aurélio Raeder da Costa, and Júlio Cezar Uili Coelho declare that they have no conflict of interest and that the ethical principles were followed.

Ethics approval: This study complies with institutional/national ethical standards and was approved by Hospital Nossa Senhora das Graças. There is no need for evolution by National Research Ethics Commission (Plataforma Brasil) to short report.

Humans and animal rights: This article does not contain any study with animals performed by any of the authors.
